# Creation of Artificial Luciferase 60s from Sequential Insights and Their Applications to Bioassays

**DOI:** 10.3390/s23146376

**Published:** 2023-07-13

**Authors:** Sung-Bae Kim, Tadaomi Furuta, Nobuo Kitada, Shojiro A. Maki

**Affiliations:** 1 Research Institute for Environmental Management Technology, National Institute of Advanced Industrial Science and Technology (AIST), Tsukuba 305-8569, Japan; 2 School of Life Science and Technology, Tokyo Institute of Technology, Yokohama 226-8501, Japan; furuta@bio.titech.ac.jp; 3 Department of Engineering Science, Graduate School of Informatics and Engineering, The University of Electro-Communications, Chofu 182-8585, Japan; kitada@uec.ac.jp (N.K.); s-maki@uec.ac.jp (S.A.M.)

**Keywords:** artificial luciferase, bioluminescence, coelenterazine, copepod luciferase, single-chain bioluminescent probe, molecular strain probe

## Abstract

In this study, a series of new artificial luciferases (ALucs) was created using sequential insights on missing peptide blocks, which were revealed using the alignment of existing ALuc sequences. Through compensating for the missing peptide blocks in the alignment, 10 sibling sequences were artificially fabricated and named from ALuc55 to ALuc68. The phylogenetic analysis showed that the new ALucs formed an independent branch that was genetically isolated from other natural marine luciferases. The new ALucs successfully survived and luminesced with native coelenterazine (nCTZ) and its analogs in living mammalian cells. The results showed that the bioluminescence (BL) intensities of the ALucs were interestingly proportional to the length of the appended peptide blocks. The computational modeling revealed that the appended peptide blocks created a flexible region near the active site, potentially modulating the enzymatic activities. The new ALucs generated various colors with maximally approximately 90 nm redshifted BL spectra in orange upon reaction with the authors’ previously reported **1**- and **2**-series coelenterazine analogs. The utilities of the new ALucs in bioassays were demonstrated through the construction of single-chain molecular strain probes and protein fragment complementation assay (PCA) probes. The success of this study can guide new insights into how we can engineer and functionalize marine luciferases to expand the toolbox of optical readouts for bioassays and molecular imaging.

## 1. Introduction

Bioluminescence (BL) is an emerging optical readout in various bioassays and molecular imaging modalities, which is generated through the catalytic reaction of luciferase with its specific substrate. BL was observed in a large variety of luminous insects, marine organisms, and prokaryotes in nature [1].

To date, many marine luciferases from sea organisms have been identified, such as the sea pansy *Renilla reniformis* [2], the copepods *Gaussia princeps* [3] and *Metridia longa* [4], the ostracod *Cypridina noctiluca* [5], the dinoflagellate *Pyrocystis lunula* [6], and the deep sea shrimp *Oplophorus gracilirostris* [7]. These marine luciferases are categorized into non-secretory and secretory luciferases. Copepod luciferases among the marine luciferases are members of a family of secretory proteins derived from marine copepods. Copepod luciferases are attractive reporters due to their small size, strong BL, and high thermostability [8].

However, BL from natural marine luciferases is not necessarily optimal as an optical readout because it is simply an accidental consequence of Darwinian evolution under selection pressures [9,10].

Conventionally, the optical properties of BL were improved through mutagenesis studies on the sequences of the luciferases [11]. This mutagenetic approach was practically successful in previous studies on the variants of firefly luciferase (Akaluc) [12] and *Renilla* luciferase (RLuc) [11]. *Gaussia* luciferase (GLuc), Metridia pacifica luciferase 1 (MpLuc1), and Metridia longa luciferase (MLuc) were also genetically mutated to improve the optical properties, such as the BL intensity and stability [13]. However, this is often very slow, tedious, and labor-intensive. Moreover, structural information on many luciferases by experiments is still not known or rarely available [14]. Alternatively, a new strategy was previously developed to create artificial luciferases (ALucs) by extracting a whole sequence of the consensus amino acids (AAs) from the alignment of many natural copepod luciferases available in the public databases, for example, the National Center for Biotechnology Information (NCBI) database [15,16]. The theoretical background of this approach was postulated on the premise that the frequently occurring AAs at a given position have a larger thermostabilizing effect compared with the less frequent ones and are a genetically evolved property. This approach was surprisingly successful in the fabrication of a lineage of ALucs and found that the sequences are phylogenetically distinctive from any existing marine luciferases [15,16].

**Figure 1:**
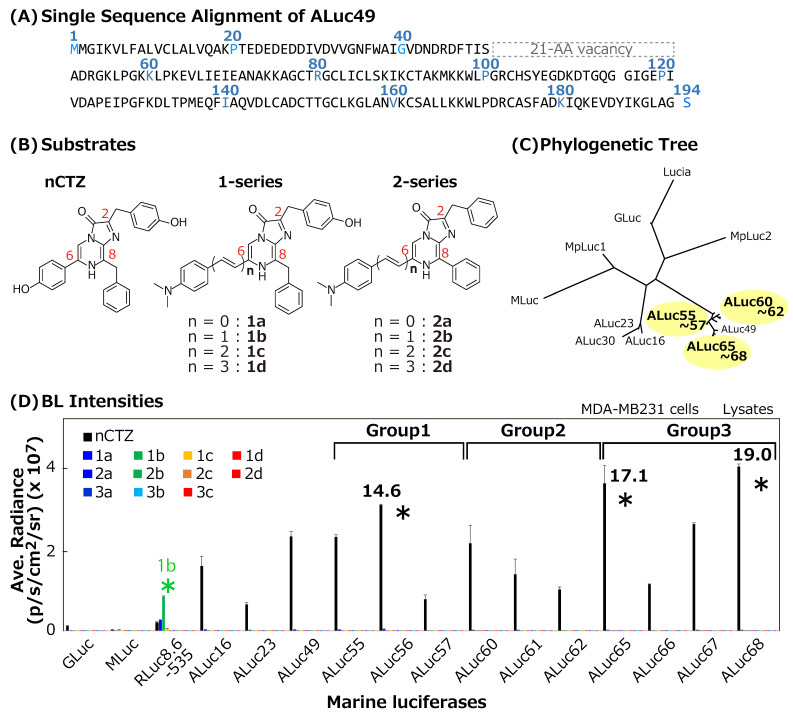
(**A**) Single-sequence alignment (SSA) of artificial luciferase 49 (ALuc49) as a primary sequence of new artificial luciferases. Three split regions are represented in each row. (**B**) The chemical structures of the native coelenterazine (nCTZ) and representative CTZ analogs. (**C**) Phylogenetic tree of new ALuc series luciferases compared with conventional ones. The yellow shadow highlights the relative positions of new artificial luciferases. (**D**) Comparison of the absolute BL intensities of new ALuc series luciferases with **1**- and **2**-series CTZ analogs as substrates. The numbers in the bar graphs denote the fold intensities relative to that of RLuc8.6-535. The asterisks highlight the BL signals with prominent optical intensity and specificity.

Although the previous extraction-based approaches were successful, the authors noted that these smallest luciferases from copepods are interestingly aligned into three homological regions, which may hide secrets about their unique optical properties. Inspired by this unique feature, the authors speculated whether new competent sibling luciferases can be fabricated from these sequential insights. Because typical copepod luciferases consist of two repeated, conserved catalytic domains and one flexible N-terminal region [17,18], these sequences can be split into three and aligned with respect to the sequential homology. This alignment revealed vacancy in the N-terminal region (Figure 1). Furthermore, the authors hypothesized whether new sibling sequences can be made by compensating the vacant region (missing peptidesite) in the sequence with respect to the improvement of the consensus AAs. This approach is adequate to investigate the unknown contribution of the N-terminal region to the enzymatic properties. Therefore, 10 sibling sequences were artificially created and named from ALuc55 to ALuc68. The new ALucs were characterized in terms of the absolute BL intensity, the BL spectrum and color, and the substrate specificity. The optical properties were interpreted with putative three-dimensional (3D) conformations of the new ALucs. Moreover, the advantages of the new ALucs were further demonstrated with the sensorial utilities of new single-chain molecular strain probes and protein fragment complementation assay (PCA) probes. To date, many protein–protein interaction (PPI) models have been reported using ALucs and single-chain probe modalities, which include androgen receptor (AR)–LXXLL motif binding [19], glucocorticoid receptor (GR)–LXXLL motif binding [20], and FRB–FKBP binding [21]. The phosphorylation of the tyr537 of ER was also reported by previous papers [22,23].

All studies demonstrated that new ALucs can indeed be created using alignment insights and successfully utilized as optical readouts in various bioassay probes in mammalian cells. Considering that the present strategy can be applicable to other luciferase strains, this study guides new methodology on how we can expand the toolbox of optical readouts and functionalize them in bioassays and molecular imaging.

## 2. Materials and Methods

### 2.1. Design of New ALucs Using Single-Sequence Alignment (SSA)

As a template of single-sequence alignment (SSA), the sequence of ALuc49 was chosen and conceptionally fragmented into three regions. They were then aligned using the web software CLUSTALW version 2.1. Because the alignment revealed a vacant site (gap) at the top right region, it was supplemented with the amino acid (AA) blocks “^51^GRCHSYEG^58^,” “^51^GRCHSYEG DKDTGQG^65^,” and “^51^GRCHSYEG DKDTGQG GIGEPI^71^” (Supplementary Materials Figures S1 and S2), where the AA blocks were decided on in a way to mimic the corresponding AA sequences in the other regions revealed by the SSA. Upon deciding on the individual AA block, the corresponding AA sequence was conceptually divided into several fragments (blocks) near the flexible amino acids, such as glycine (G), isoleucine (I), alanine (A), or valine (V). They were named “amino acid block” or simply “peptide block” in this study. The consequent sequences were named ALuc55, ALuc56, and ALuc57, respectively. In parallel, the vacant site (gap) in the alignment of the same prototypical sequence ALuc49 was filled up with the AA blocks “^51^DRCASFA^57^”, “^51^DRCASFA DKIQKEV^64^”, and “^51^DRCASFA DKIQKEV DYIKGLAG^72^”, and named ALuc60, ALuc61, and ALuc62, respectively. Additionally, the original AA sequence “^47^FTIS^50^” of ALuc49 was replaced with “^47^KWLP^50^”, which was then named ALuc65. The vacant site (gap) in the alignment of ALuc65 was further filled up with the AA sequences “^51^GRCHSYEG^58^”, “^51^GRCHSYEG DKDTGQG^65^”, and “^51^GRCHSYEG DKDTGQG GIGEPI^71^”, which were then named ALuc66, ALuc67, and ALuc68, respectively. The C-terminal ends of all the original and AA-complemented ALuc sequences were then tagged with the endoplasmic reticulum (ER) retention signal “KDEL” for their retention in the ER. The tag “KDEL” was used in this study because it is the most common peptide signal that retains the host proteins in the ER compartment [24], where the host proteins are recognized by endogenous KDEL receptors and retained in the ER.

The average AA length and molecular weight (MW) of the newly made ALucs were 195.7 (±5.7 s.d.) AAs and 21.2 (±0.7 s.d.) kD, respectively. The average theoretical isoelectric point (pI) was found to be 5.7 (±0.5 s.d.).

### 2.2. Fabrication of cDNA Constructs Encoding the New ALucs

All artificially designed AA sequences (Supplementary Materials Figure S1) were converted to murine-codon-optimized cDNA sequences using web software version 2.1 provided by Eurofins Genomics (Tokyo, Japan). The cDNA sequences encoding the ALucs were custom-synthesized on order by Eurofins Genomics. The synthesized cDNAs were then subcloned into a mammalian expression vector pcDNA3.1(+) (Invitrogen, MA, USA) using the specific restriction sites *Hind*III and *Xho*I. The overall sequence fidelity was confirmed using a sequencing service provided by Eurofins Genomics.

### 2.3. Determination of the Phylogenetic Tree and Identity of the New ALucs

The phylogenetic tree and identity of the artificially designed ALucs were determined to confirm the sequential originality from conventional marine luciferases (Figure 1C).

The amino acid sequence information of conventional copepod luciferases besides the present ALuc sequences was collected from our previous studies [15,16]. The unrooted phylogenetic trees of the artificially designed ALucs and existing marine luciferases were determined using the web software CLUSTALW version 2.1. In parallel, we searched for the sequential identity of the selected sequences of the new ALucs (i.e., ALuc56, ALuc65, and ALuc68) using the web software BLAST version 2.13.0+ of the National Center for Biotechnology Information (NCBI). The top-ranked sequences were listed in Supplementary Materials Table S1.

### 2.4. Determination of the Absolute BL Intensities of the New ALucs and Conventional Marine Luciferases

The BL intensities of the new ALucs were examined in MDA-MB231 cells (MDA cells hereafter) derived from human breast cancer (Figure 1D).

MDA cells were grown in 6-well microplates with Dulbecco’s minimal essential medium (DMEM; high glucose), supplemented with 10% (*v*/*v*) heat-inactivated fetal bovine serum (FBS) and 1% (*v*/*v*) penicillin/streptomycin (P/S; Thermo Fisher Scientific, MA, USA). The MDA cells were transiently transfected with a pcDNA3.1(+) vector, encoding each ALuc or conventional marine luciferase, that is, *Gaussia princeps* luciferase (GLuc; GenBank AAG54095.1) [25], *Metridia longa* luciferase (MLuc), or *Renilla reniformis* luciferase 8.6-535 (RLuc8.6-535) [11], as an internal reference using the lipofection reagent TransIT-LT1 (Mirus). The cells in the 6-well microplates were incubated overnight in a humidified 5% (*v*/*v*) CO_2_ incubator. The cells were then subcultured in 96-well black frame microplates. In this experiment, the expression levels of the luciferases were not controllable. To tackle this limitation, we simultaneously determined a series of internal references in parallel, that is, multiple conventional luciferases, GLuc, MLuc, and ALuc16.

After overnight incubation, the cells in each well were passively lysed with 40 μL of a lysis buffer (E291A; Promega, WI, USA) according to the manufacturer’s instructions. The relative BL intensities in the microplate were simultaneously determined using an IVIS Spectrum imaging system (PerkinElmer, MA, USA) immediately after simultaneous injections of 40 μL of the PBS buffer containing a native coelenterazine (nCTZ), **1a**, **1b**, **1c**, **1d**, **2a**, **2b**, **2c**, **2d**, **3a**, **3b**, or **3c** substrate using a multichannel micropipette, where the 1- and 2-series coelenterazine (CTZ) analogs were obtained from our previous study [26] (Figure 1D).

In this study, nCTZ and its analogs, such as **1a**–**d** and **2a**–**d**, were used for the BL development because CTZ is the common substrate for marine luciferases.

### 2.5. Structural Modeling of New ALucs for Locating the Variable Loop Regions

**Figure 2:**
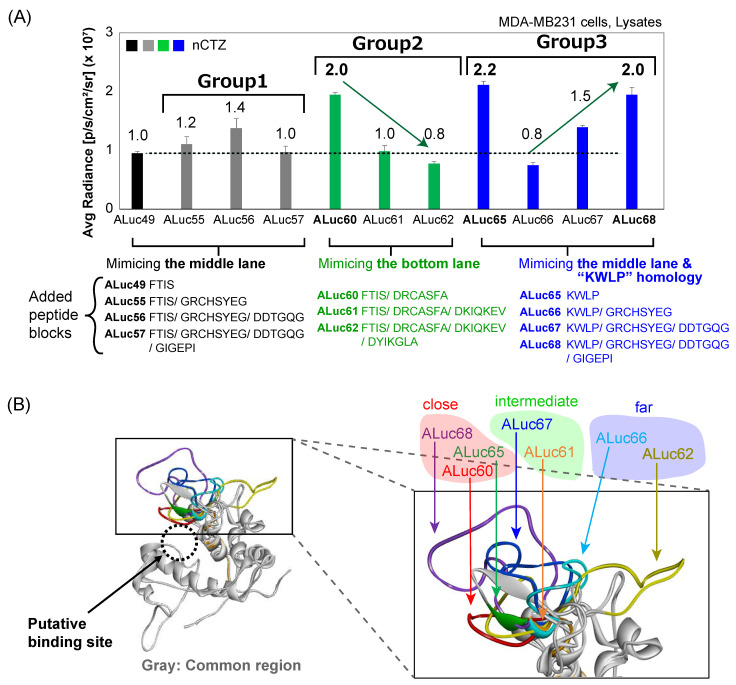
(**A**) Comparison of the BL intensities of the new ALucs, which were categorized into three groups. The numbers on the bar graphs show the fold intensity of each ALuc compared with that of ALuc49. (**B**) The three-dimensional (3D) model structures of the new ALucs. The conserved regions among the new ALucs are shown in gray, whereas the variable loop regions are highlighted in colors. The magnified residues in the variable loop regions of ALuc60, ALuc65, and ALuc68 were close to the substrate binding site.

The BL intensity variations of the new Alucs (ALuc60–68) depended on the variable loop regions between them (Figure 2A). Therefore, structural modeling was conducted (Figure 2B).

The amino acid sequences of these new ALucs are shown in Supplementary Materials Figure S1. Using these sequences, the 3D model structures of the new ALucs were created based on the homologous *Gaussia* luciferase (GLuc) NMR structure [27] as a template from SWISS-MODEL (https://swissmodel.expasy.org (accessed on 1 September 2022)) [28]. The pairwise alignments of each ALuc and template (GLuc) in SWISS-MODEL depended on hidden Markov models (HMM), and thus, they did not necessarily match the alignment gaps in Supplementary Materials Figure S1, leading to the displacement of the colored regions in Figure 2B. In principle, template-based modeling, such as that undertaken with SWISS-MODEL, requires a reliable template structure. Unfortunately, none of the ALuc structures had been experimentally analyzed yet according to Protein Data Bank (RCSB, USA). Hence, the existing GLuc structure with the highest homology was used here as the template.

### 2.6. Determination of the BL Spectra of the New ALucs

**Figure 3:**
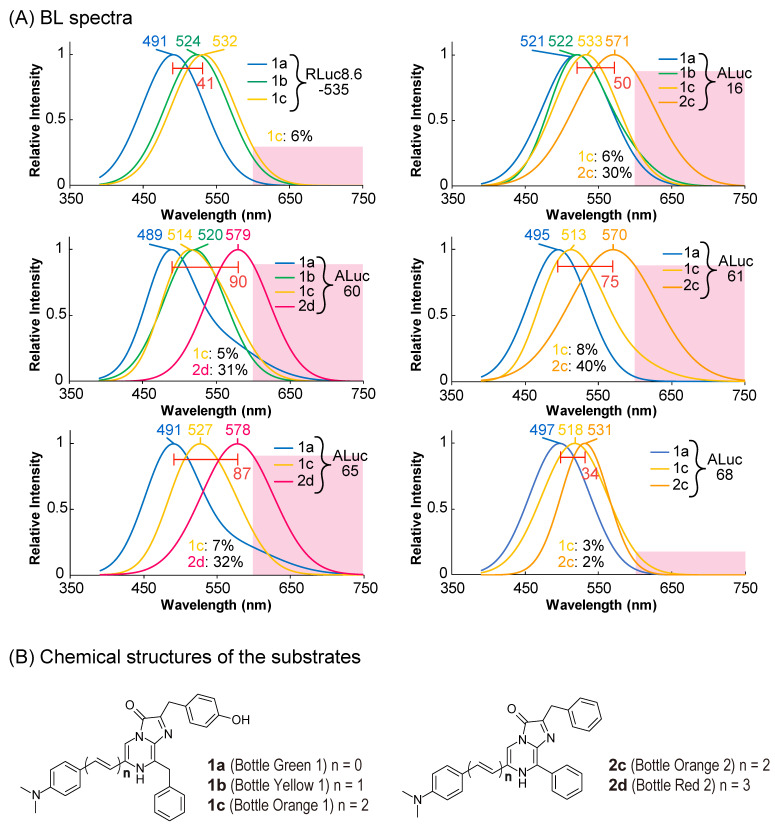
(**A**) BL spectra of RLuc8.6-535 and the new ALuc series luciferases according to the **1**- and **2**-series CTZ analogs. The pink shadows highlight wavelengths longer than 600 nm. (**B**) The chemical structures of the **1**- and **2**-series CTZ analogs that were used in the measurement of the BL spectra.

The colorimetric variety of the new ALucs with various CTZ analogs was investigated in MDA cells (Figure 3).

MDA cells containing the new ALucs or Rluc8.6-535 were prepared via lipofection using the same method used in Figure 1D. The cells were passively lysed with a lysis buffer (Promega) according to the manufacturer’s instructions. The lysates, 40 μL each, were aliquoted into 200 μL PCR tubes, and each tube was injected with 40 μL of PBS, dissolving each substrate. The tube was immediately mounted in the sample stage of a spectrophotometer (AB-1850, ATTO, Tokyo, Japan), and the corresponding BL spectra were determined and analyzed using the specific software LumiFLSpectroCapture version 1.0 and IGOR Pro version 7 (WaveMetrics, Portland, OR, USA).

### 2.7. Construction of Single-Chain Probes Mimicking Molecular Strain Probes with the New ALucs

**Figure 4:**
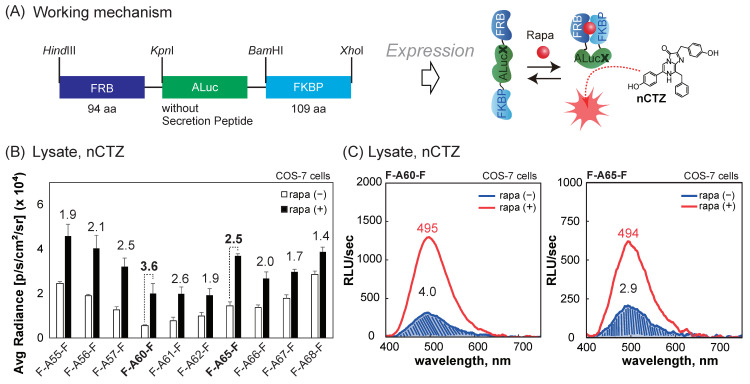
(**A**) Schematic diagram of the cDNA construct encoding a single-chain molecular strain probe. In the ALuc sequence, the secretion peptide (SP) was eliminated. The working mechanism after the expression is briefly illustrated in the right panel. Abbreviations: ALucX, any artificial luciferase; FRB, FKBP-rapamycin binding domain; FKBP, FK506-binding protein; rapa, rapamycin. (**B**) BL intensities of a series of molecular strain probes embedding ALuc55–ALuc68. Rapamycin triggered intramolecular protein–protein interactions between FRB and FKBP, which appended the molecular strain to the sandwiched ALuc. This strain boosted the BL intensities of the sandwiched ALuc. (**C**) The BL spectra of F-A60-F and F-A65-F in the presence or absence of rapamycin.

The utilities of the newly fabricated ALucs in bioassays were determined using single-chain probes mimicking molecular strain probes carrying full-length ALucs (Figure 4).

The single-chain probes were genetically designed and synthesized as follows: cDNA encoding the full-length ALuc55–68, except the N-terminal secretion peptide (SP) was genetically synthesized via polymerase chain reaction (PCR) to introduce the specific restriction sites *Kpn*I and *Bam*HI at the 5′ and 3′ ends; the cDNA fragments encoding FKBP-rapamycin binding domain of FKBP12-rapamycin associated protein (FRB) and FK506-binding protein (FKBP) were similarly made using PCR to introduce *Hind*III/*Kpn*I and *Bam*HI/*Xho*I sites at the 5′ and 3′ ends, respectively; the cDNA fragments were digested by the corresponding restriction enzymes, ligated in the order of FRB–ALuc–FKBP, and subcloned into pcDNA3.1(+) vectors; the fidelity of the cDNA constructs was confirmed using a genetic sequence analyzer by order (Eurofin genomics); and the created single-chain probes were named from F-A55-F to F-A68-F based on the sandwiched ALuc name.

The assays, using the single-chain probes, were conducted through the following procedure (Figure 4B,C). COS-7 cells were grown in 6-well microplates to reach 80% confluency. The cells in each well were transiently transfected in order with a pcDNA3.1(+) vector, encoding one of the following probes using the lipofection reagent, namely, F-A55-F, F-A56-F, F-A57-F, F-A60-F, F-A61-F, F-A62-F, F-A65-F, F-A66-F, F-A67-F, or F-A68-F. The cells were incubated overnight and subcultured into a 96-well black frame microplate. The cells were then stimulated overnight with 10^−6^ M rapamycin or vehicle (0.1% (*v*/*v*) alcohol) in a culture medium. After the elimination of the culture medium, the cells were lysed with 40 μL of a lysis buffer (Promega) per well according to the manufacturer’s protocol. The lysates in the wells were simultaneously injected with 40 μL of the substrate solution containing nCTZ using a multichannel micropipette. The corresponding BL images were immediately determined using an IVIS Spectrum imaging system (PerkinElmer).

In parallel, 60 μL of the lysates with rapamycin or the vehicle were aliquoted into 200 μL PCR tubes and injected with 60 μL of the substrate solution containing nCTZ. The mixture was immediately mounted on the sample stage of a spectrophotometer (AB-1850, ATTO), and the corresponding BL spectra were determined and analyzed using specific software LumiFLSpectroCapture version 1.0 and IGOR Pro version 7 (WaveMetrics).

### 2.8. Protein Fragment Complementation Assay (PCA) Systems for Determining Sex Hormones

**Figure 5:**
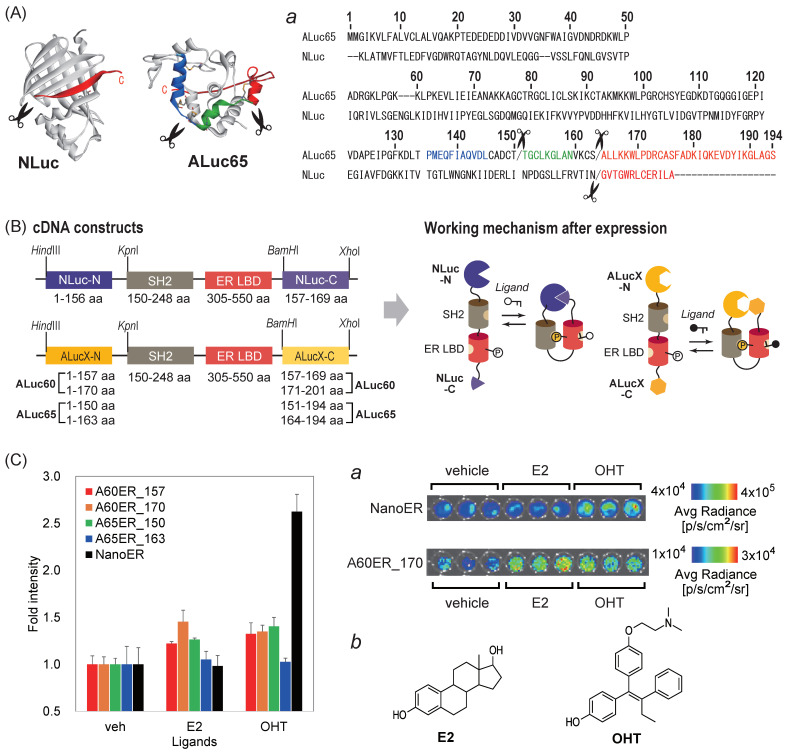
Applications of ALuc60 and ALuc65 to protein fragment complementation assay (PCA) systems. (**A**) The three-dimensional (3D) structures of NLuc (PDB: 5IBO) and the putative structure of ALuc65. Inset ***a*** shows the sequences of NLuc and ALuc65. The scissors marks denote the dissection sites in this study. The putative α-helices near the dissection sites of ALuc65 were highlighted in blue, green, and red. (**B**) Schematic diagram of the cDNA constructs encoding the PCA probes and the working mechanism after expression. (**C**) The BL intensities of various single-chain PCA probes in response to estrogen agonist 17β-estradiol (E2) and antagonist 4-hydroxytamoxifen (OHT). Inset ***a*** shows the corresponding BL images. Inset ***b*** illustrates the chemical structures of E2 and OHT.

New single-chain bioluminescent probes for determining sex hormones were developed based on the concept of the PCA (Figure 5).

First, the cDNA constructs were designed according to the following methods: cDNA fragments encoding the N- and C-terminal fragments of NLuc (1–156/157–169 AA, where the slash means the dissection point) were generated using PCR to introduce *Hind*III/*Kpn*I and *Bam*HI/*Xho*I sites, respectively. The dissection site was referenced from a previous study [29]. The cDNA fragments encoding the Src SH domain (150–248 aa)-linked human estrogen receptor ligand-binding domain (ER LBD; 305–550 AA) was obtained from our previous study [30] and genetically ligated between the cDNA fragments encoding the N- and C-terminal fragments of NLuc using the *Kpn*I and *Bam*HI sites as shown in Figure 5. The cDNA construct was subcloned into a pcDNA3.1(+) vector (Invitrogen). The probe after the mammalian expression was named NanoER.

Separately, cDNA fragments encoding the N- and C-terminal fragments of ALuc60 (1–157/158–201 AA or 1–170/171–201 AA) and ALuc65 (1–150/151–194 AA or 1–163/164–194 AA) were made using PCR to introduce *Hind*III/*Kpn*I and *Bam*HI/*Xho*I sites, respectively. The cDNA fragments encoding the same Src SH domain-linked ER LBD were obtained from our previous study [30] and genetically ligated between the cDNA fragments encoding the N- and C-terminal fragments of ALucs using the *Kpn*I and *BamH*I sites, as shown in Figure 5. The cDNA construct was subcloned into a pcDNA3.1(+) vector (Invitrogen, MA, USA). The probes after expression were named A60ER_157, A60ER_170, A65ER_150, and A65ER_163, respectively, wherein the first and second numbers denote the incorporated ALuc and the dissection site, respectively. The overall sequence fidelity was confirmed using a sequencing service provided by Eurofins Genomics (Tokyo, Japan).

The constructed probes were used for the determination of sex hormones through the following protocol: COS-7 cells grown in the wells of a 6-well microplate were transiently transfected with one of the pcDNA3.1(+) vectors encoding NanoAR, A60ER_157, A60ER_170, A65ER_150, or A65ER_163, as well as A65ER (0.1 μg per well) using a lipofection reagent (TransIT-LT1, Mirus). Two days after the transfection, the cells were subcultured into 96-well black frame microplates and incubated for another day. The cells were then stimulated for 20 min with a vehicle (culture medium comprising 0.1% DMSO), 10^−6^ M of 17β-estradiol (E2), or 10^−6^ M of DHT.

Before the BL measurements, the cell culture media were thoroughly eliminated, and the remaining cells were passively lysed with a cell lysis buffer (Wako Pure Chemical, Tokyo, Japan) for 15 min. The wells were then simultaneously injected with 40 μL of the PBS buffer containing nCTZ or other substrates using a multichannel micropipette. The corresponding BL intensities were determined using an IVIS Spectrum imaging system (PerkinElmer).

## 3. Results and Discussion

### 3.1. New ALucs Could Be Constructed to Fill the Vacant Region in the Blocks

Copepod luciferases consist of two conserved domains, which have a high homology and a flexible N-terminal region [17,18]. In this study, the sequence was aligned into three-story rows to match the homological amino acids (AAs), revealing the deficient peptide blocks. As shown in Figure 1A, the alignment revealed a uniquely vacant region around the 50th AA, which was compensated for with a series of AA blocks to enhance the consensus AAs among the sequences to create new sibling ALucs (Supplementary Materials Figures S1 and S2), where the term “sibling” was used given that the new ALucs are similar to the previous ALuc series but not the same.

The new ALucs were found to be phylogenetically distinctive from other marine luciferases based on the phylogenetic tree analysis and homology search (Figure 1C and Supplementary Materials Table S1). In the analysis, the most similar sequence to both ALuc56 and ALuc65 was that of the synthetic Mluc7 construct from *Metridia longa*, which had maximal identity scores of approximately 76.4 and 77.2%, respectively. In the case of ALuc68, the most similar sequence was that of *Metridia pacifica* luciferase (MpLuc), with an identity score of 79.3%. In parallel, the phylogenetic tree shows that the new ALucs formed an independent branch, which was isolated from conventional natural copepod luciferases and our previous ALuc series, such as ALuc16, ALuc23, and ALuc30 (Figure 1C).

### 3.2. New ALucs Survived in Mammalian Cells and Emitted Strong BL

It is an important criterion to determine whether the newly designed ALucs are properly expressed and survive and emit BL in the context of mammalian cells.

Hence, the absolute BL intensities were determined to clarify (i) whether the new ALucs were properly expressed and survived and preserved the enzymatic activities in mammalian cells, and (ii) whether the compensation of the missing peptide blocks to increase the homology among the aligned sequences practically contributed to the BL intensities and/or color variation because it is empirically known that higher homology allows for stronger BL intensities [15,31].

The results in Figure 1D show that all new ALucs were successfully expressed and luminesced with nCTZ in MDA-MB231 cells. The overall BL intensities of the new ALucs with nCTZ were brighter than those of natural copepod luciferases, such as GLuc and MLuc. Among the tested substrates, ALuc series luciferases were highly selective for nCTZ, whereas RLuc8.6-535 was the brightest with **1b**, followed by **1a** and nCTZ. RLuc8.6-535 was bright enough with nCTZ, but simply appeared dim upon comparison with the other substrates under the experimental condition using the Promega lysis buffer. In the presence of nCTZ, the new ALucs, namely, ALuc56, ALuc65, and ALuc68, were approximately 14.6-, 17.1-, and 19.0-fold brighter than RLuc8.6-535, respectively.

The new ALucs were categorized into three groups based on the sequential similarity of the compensated blocks, that is, ALuc55–57, ALuc60–62, and ALuc65–68 (Figure 1D). Because each group in Figure 1D revealed that the BL intensities were influenced by the compensated lengths of AAs, the BL intensity profiles were further highlighted in detail (Figure 2A and Supplementary Materials Figure S3).

The peptide blocks in the top lane of group 1 were compensated by filling them with the corresponding AAs of the middle lane in the alignment, whereas the appended blocks in group 2 were compensated by filling them with the sequence at the bottom lane. The BL intensity of the ALucs in group 1 weakly increased with ALuc56 and then returned to the basal value by elongating the compensation of the missing peptide blocks. However, in group 2, a significant drop in the BL intensities was observed because the compensated peptide was elongated. In group 3, the intensities dropped with ALuc66 but recovered with ALuc67 and ALuc68 because the compensated peptide length became longer.

### 3.3. The Variable Loop Regions of the New ALucs Dominated the BL Intensities

Because Figure 2A reveals that the BL intensities were influenced by the length of the compensated peptides, the structural models of the new ALucs (ALuc60–68) were investigated using SWISS-MODEL (https://swissmodel.expasy.org (accessed on 1 September 2022)) [28], where the compensated peptides are highlighted in colors (Figure 2B).

These models indicated that the compensated peptides in the top domain formed flexible loops instead of rigid α-helical or β-sheet structures. In addition, the 3D structure revealed that the variable loop regions of the higher BL intensity ALucs (ALuc60, ALuc65, and ALuc68) closely covered the substrate binding site, whereas the others were located in the middle range (ALuc61 and ALuc67: middle BL intensity) or the far range (ALuc62 and ALuc66: low BL intensity) from the substrate binding site.

As a reference index, the inter-side chain distances between the Met adjacent to the binding site and the nearby residues in the loops were found to be as follows: i.e., ALuc60—3.4 Å (F56–M143), ALuc65—5.7 Å (W48–M136), and ALuc68—5.8 Å (E69–M157) as close-distance cases of 3–6 Å; ALuc61—6.8 Å (D58–M150) and ALuc67—8.0 Å (D46–M151) as middle-distance cases of 7–8 Å; and ALuc66—8.7 Å (D46–M144) and ALuc62—5.7 Å (I67–M158) as far-distance cases of ~9 Å (note that the main part of the loop of ALuc62 was located on the far side). All these data suggest a close correlation between the BL intensity and loop conformation. Although these variable loop regions might be flexible in an aqueous phase, the basic conformations covering the substrate binding site should be relevant for substrate association, which influences the BL intensity.

This relationship between the location of the variable loop regions and the tendency of the BL intensity might provide important insights into the catalytic mechanism of luciferases. Currently, AlphaFold [32], which is a state-of-the-art technology, is effective for various modeling, but modeling of variable loop parts in these flexible enzymes is uncertain, and using SWISS-MODEL would be considered sufficient for this study. Follow-up studies regarding the insights found should allow for a better understanding of the enzyme–substrate relationship.

### 3.4. Redshifted BL Spectra of the New ALucs with Coelenterazine Analogs

Because some of the new ALucs were relatively bright, their BL spectra were further characterized with various coelenterazine (CTZ) analogs previously reported by the authors in the **1**- and **2**-series [26] (Figure 3 and Table 1).

Among the tested luciferases, the following emitted BL spectra with remarkable intensities, and thus, are highlighted in Figure 3A: ALuc16, ALuc60, ALuc61, ALuc65, and ALuc68, besides RLuc8.6-535. The spectral peaks are summarized in Table 1.

The CTZ analog **2d** commonly generated the most redshifted BL spectra with the marine luciferases. For example, **2d** was yellowish-orange and peaked at 579 and 578 nm with ALuc60 and ALuc65, respectively. Their BL spectral portions longer than 600 nm were approximately 30%, which allow high tissue permeability in animal models. The CTZ analog **2c** also showed largely redshifted BL spectra that peaked at 571 and 570 nm with ALuc16 and ALuc61, respectively. Their BL spectral portions longer than 600 nm were found to be 30–40%. However, the most blue-shifted spectra were commonly obtained with the **1a** substrate, for example, **1a** emitted bluish-green BL spectra that peaked at 489 and 491 nm with ALuc60 and ALuc65, respectively. Their BL spectral portions longer than 600 nm were merely 8%.

In the case of ALuc60, the colors could be modulated from bluish-green to orange by simply changing the substrate from **1a** to **2d**. The gap between the peak gaps was approximately 90 nm. In addition, the color of ALuc65 was variable from bluish-green to orange by replacing the substrates **1a** with **1c** or **2d**. The peak gap between the colors was found to be approximately 87 nm. The overall optical properties of ALuc60 and ALuc65 are compared with conventional marine luciferases in Table 2.

Conventionally, the BL colors were explained to have differences in the energy levels of the intermediates of the substrates during the catalytic reaction with a luciferase [33,34]. The mechanism may be explained as follows: In the case of nCTZ, it shows a broad emission peak from blue to green (400–535 nm) because of four different energy levels of intermediates, that is, neutral species, amide anion, phenolate anion, and pyrazine anion [35]. The neutral species is attributed to blue, and the pyrazine anion species generates the most redshifted color. In the present study, the most redshifted spectrum peak was found at 579 nm with the ALuc60–**2d** combination. This redshifted feature longer than 535 nm may be explained by the elongated π-conjugation at the C-6 position of **2d**, which was much longer than that of nCTZ. **2d** should be converted to π-conjugation-elongated intermediates through the catalytic reaction with ALuc60 or ALuc65, resulting in redshifted BL emission.

**Table 2:** Specification of conventional marine and artificial luciferases that were used in this study.

Luciferase (Abbreviation) (MW)	Origin (λ_max_, nm ^a^)	Substrate	Features
*Renilla* luciferase (RLuc) (36 kD) [2]	*Renilla reniformis* (sea pansy) (480 nm)	CTZ	Thermolabile
*Renilla* luciferase 8.6-535 (RLuc8.6-535) (36 kD; Mut. of RLuc) [11]	*Renilla reniformis * (sea pansy) (535 nm)	CTZ	Bright and red-shifted
*Gaussia* luciferase (GLuc) (20 kD) [3]	*Gaussia princeps* (copepod) (470 nm)	CTZ	Secreted
NanoLuc (NLuc) (19 kD; Mut. of a small subunit of OLuc) [36]	*Oplophorus gracilorostris* (deep sea shrimp) (456 nm)	Furimazine	High pH and thermostability, very bright, and stable and prolonged light emission
*Metridia longa* luciferase (MLuc) (24 kD) [4]	*Metridia longa* (copepod) (480 nm)	CTZ	Secreted and small molecular weight
Artificial Luciferase 16 (ALuc16) (23 kD) [15]	*Copepoda luciferase database* (496 nm)	CTZ	Secreted, bright, and λ_max_ was variable according to buffer conditions
Artificial Luciferase 23 (ALuc23) (23 kD) [15]	*Copepoda luciferase database* (503 nm)	CTZ	Secreted, bright, and strain-sensitive
Artificial Luciferase 49 (ALuc49) (21 kD) [16]	*Copepoda luciferase database* (490 nm)	CTZ	Secreted and specific to CTZ
Artificial Luciferase 60 (ALuc60) (22 kD)	*Modified from ALuc49* (497 nm with nCTZ; 579 nm with 2d)	CTZ	Secreted and bright
Artificial Luciferase 65 (ALuc65) (21 kD)	*Modified from ALuc49* (492 nm with nCTZ; 578 nm with 2d)	CTZ	Secreted and bright

^a^ Maximum wavelength of bioluminescence spectrum in nanometers. Mut. denotes mutant.

### 3.5. The New ALucs Were Useful Optical Reporters for Single-Chain Probes Mimicking Molecular Strain Probes

The utilities of the new ALucs as optical reporters were demonstrated with single-chain probes mimicking molecular strain probes (Figure 4).

The basic concept of single-chain molecular strain probes was originally established by the authors for the first time [21,37,38]. In the molecular design, a full-length luciferase was sandwiched between two proteins of interest. Once the two proteins created an intramolecular protein–protein interaction, the sandwiched luciferase may be strained to modulate the enzymatic activities.

In this study, all the new ALucs were genetically sandwiched between FRB and FKBP and created new single-chain probes that mimicked the preceding molecular strain probes (Figure 4A). The secretion peptides (SPs) at the N-terminal end were eliminated beforehand because our previous study showed that SPs hamper the proper working of the probes [38]. The results showed that all the single-chain probes enhanced the BL intensities in a rapamycin-dependent manner (Figure 4B). The highest S/B ratios were obtained using F-A60-F with 3.6-fold and using F-A65-F with 2.5-fold, where F-AX-F stands for FRB-ALucX-FKBP and its capital “X” means the series number of the ALuc. The highest S/B ratio of F-A60-F was aided by the lowest background BL intensity in the basal condition among the made probes.

New luciferases, namely, ALuc60, ALuc65, and ALuc68, showed relatively strong BL intensities, as seen in Figure 1A. However, the single-chain probe embedding ALuc68 showed relatively poor optical performance, as seen in Figure 4B. This result may be interpreted in terms of the longer flexible region of ALuc68 at the N-terminal end, which should relieve the molecular tension appended by the intramolecular PPI between FRB and FKBP.

We further investigated the BL spectral variance of the probes (F-A60-F and F-A65-F) in the presence or absence of rapamycin (Figure 4B). The results showed that rapamycin-stimulated F-60-F elevated the BL intensity up to 4-fold over the background. F-65-F with rapamycin stimulation also elevated the intensity by 2.9-fold greater than that without rapamycin. The BL spectral peaks were at 494–495 nm. The peak positions reflected those of the new ALuc60 and ALuc65 and confirmed that the ligand stimulation did not trigger the spectral shifts.

### 3.6. Applications to Protein Fragment Complementation Assay (PCA) Systems

The BL intensities of the single-chain BL probes carrying split A60, A65, or NLuc were determined in the presence or absence of the ligand stimulations (Figure 5).

First, the dissection sites of ALuc60 (157/158 AA and 170/171 AA) and ALuc65 (150/151 AA and 163/164 AA) for the single-chain BL probes were determined by the sequence alignment and the putative structural information of the luciferases, as shown in Figure 5A, where the corresponding dissection sites are shown in Figure 5A. The previous information on the dissection site of NLuc [29] was referenced for the dissection site decision. Moreover, when the dissection sites were decided, we also considered the secondary structural elements in the model 3D structures of ALuc60 and ALuc65 besides the structural information of NLuc. As shown in Supplementary Materials Figure S4, NLuc had a *β*-sheet-rich structure, whereas ALuc65 mostly consisted of *α*-helices. The dissection sites were chosen in the hinge regions between the *α*-helices of ALuc60 and ALuc65, as shown by the scissors marks in Figure 5A.

The basic molecular design of the constructed single-chain BL probes (Figure 5B) was inspired by our previous studies [15,30]. In the design, the Src SH2 domain-linked ER LBD was inserted between the N- and C-terminal fragments of ALuc60, ALuc65, or NLuc. After their expression, the putative working mechanism of the single-chain BL probes is suggested as follows: The ER LBD inside the probes binds the agonist or antagonist and phosphorylates the tyr537 near the α-helix 12 of the ER LBD. The phosphorylated ER LBD is bound by the adjacent SH2 domain of Src. The intramolecular conformation changes trigger the association of the N- and C-terminal fragments and recovers the enzymatic activities. Hence, the activities of the agonist and antagonist are finally converted into the detectable BL intensities. To date, many protein–protein interaction (PPI) models have been investigated and all the results supported this single-chain working mechanism, which include androgen receptor (AR)–LXXLL motif binding [19], glucocorticoid receptor (GR)–LXXLL motif binding [20], and FRB–FKBP binding [21]. The phosphorylation of the tyr537 of ER was reported by previous studies [22,23].

The results from the single-chain BL probes (Figure 5C) are summarized as follows: NanoER efficiently produced 2.6-fold stronger BL intensities with 4-hydroxytamoxifen (OHT) than with the vehicle (0.1% DMSO). Conversely, NanoER did not show significant BL intensities with E2. However, the other ALuc-based probes, namely, A60ER_157, A60ER_170, and A65ER_150, produced relatively weak intensities in response to both E2 and OHT. A60ER_170 and A65ER_150 showed 45% and 40% stronger BL intensities with E2 and OHT, respectively, compared with the vehicle. Meanwhile, A65ER_163 did not show any considerable enhancements in the BL intensities with E2 and OHT. The fold intensities of A60ER_170 and A65ER_150 were less than that of NanoER, but could be improved via optimization of the dissection sites of the ALucs for the single-chain probes, as exemplified in our previous study [15].

The overall results demonstrated that the new ALucs can be applicable to the construction of single-chain BL probes as reporters. In addition, inside the single-chain BL probes, the fragmented ALucs can recover the enzymatic activities via intramolecular association.

## 4. Conclusions

In this study, a series of new artificial luciferases (ALucs) was created using sequential insights on missing peptide blocks revealed by the alignment of existing ALuc sequences. Through compensating for the missing peptide blocks in the alignment, 10 sibling sequences were artificially fabricated and named from ALuc55 to ALuc68. The constructed ALucs were phylogenetically distinctive from natural marine luciferases. All experiments found that the 10 new sibling ALucs could be created using the alignment insights and successfully work in mammalian cells as an optical readout. The optical properties were characterized with respect to the BL intensities and spectra and confirmed that the new ALucs luminesce with strong artificial colors ranging from blue to yellowish-orange, peaking at 579 nm. The optical utilities were specified using single-chain molecular strain probes and PCA probes. The optical properties were further discussed in view of the molecular modeling between the ALucs and CTZ analogs. The new ALucs can be applicable to the construction of single-chain BL probes as split reporters. Future studies should be directed to the development of new lineages of ALucs with better optical intensities, color variety, and stability. It would also be useful to determine the optimal dissection sites for single-chain BL probes or PCA probes for expanding the applications to bioassays. Considering that the present strategy is applicable to other enzyme strains, this study is an important addition to the toolbox of optical reporters for bioassays and molecular imaging.

## Figures and Tables

**Table 1:** The maximal BL wavelengths (_λmax_) of various marine luciferases in the presence of **1**- and **2**-series CTZ analogs. The mark “-” indicates the spectral data that were not obtained in the measurements. The numbers in parentheses show the FWHM values. The _λmax_ values for **1d**, **2a**, and **2b** are not specified because reliable spectra were not obtained in the measurements.

	nCTZ in Live cells	nCTZ in Lysate	1a	1b	1c	2c	2d
ALuc16	496 (90)	496 (89)	521 (108)	522 (101)	533 (104)	571 (66)	-
ALuc60	497 (82)	482 (90)	489 (93)	520 (98)	514 (107)	-	579 (101)
ALuc61	500 (89)	490 (90)	495 (95)	-	513 (105)	570 (138)	-
ALuc62	488 (82)	493 (88)	496 (95)	-	517 (106)	-	-
ALuc65	492 (91)	495 (90)	491 (97)	-	527 (105)	-	578 (115)
ALuc66	492 (90)	492 (89)	497 (99)	-	514 (102)	-	-
ALuc67	488 (90)	491 (89)	495 (97)	-	518 (106)	-	-
ALuc68	499 (85)	495 (90)	497 (96)	-	518 (104)	531 (77)	-
R86535	540 (113)	540 (107)	491 (101)	524 (102)	532 (103)	-	-

## Data Availability

The data presented in this study are available on request from the corresponding author.

## Supplementary Materials

The following supporting information can be downloaded from https://www.mdpi.com/article/10.3390/s23146376/s1: Supplementary Materials Figure S1. Multiple sequence alignment of the newly created ALucs; Supplementary Materials Figure S2. Single-sequence alignments (SSAs) of new ALucs to highlight the appended blocks in the sequences; Supplementary Materials Figure S3. A representative BL image of the new ALucs in the presence of native coelenterazine (nCTZ); Supplementary Materials Figure S4. Three-dimensional structure of NLuc and ALuc65 showing the *α*-helices and *β*-sheets. Supplementary Materials Table S1. Homology rankings of ALuc56, ALuc65, and ALuc68 that were searched for using NCBI BLAST. Supplementary Materials Table S2. Relative optical intensity values of the bar graphs in Figure 1D, compared to those of GLuc. The terms, “Luc” and “Sub”, denote marine luciferase and substrate, respectively.
