# Supplementary material for: Creation of Artificial Luciferase 60s from Sequential Insights and Their Applications to Bioassays
Source: Sensors (Basel). 2023 Jul 13;23(14):6376. doi: 10.3390/s23146376 (PMC10384629; doi:10.3390/s23146376)
Supplement: Supplementary file 1 [file sensors-23-06376-s001.zip › sensors-2466058-supplementary.pdf]

Suppl. Information

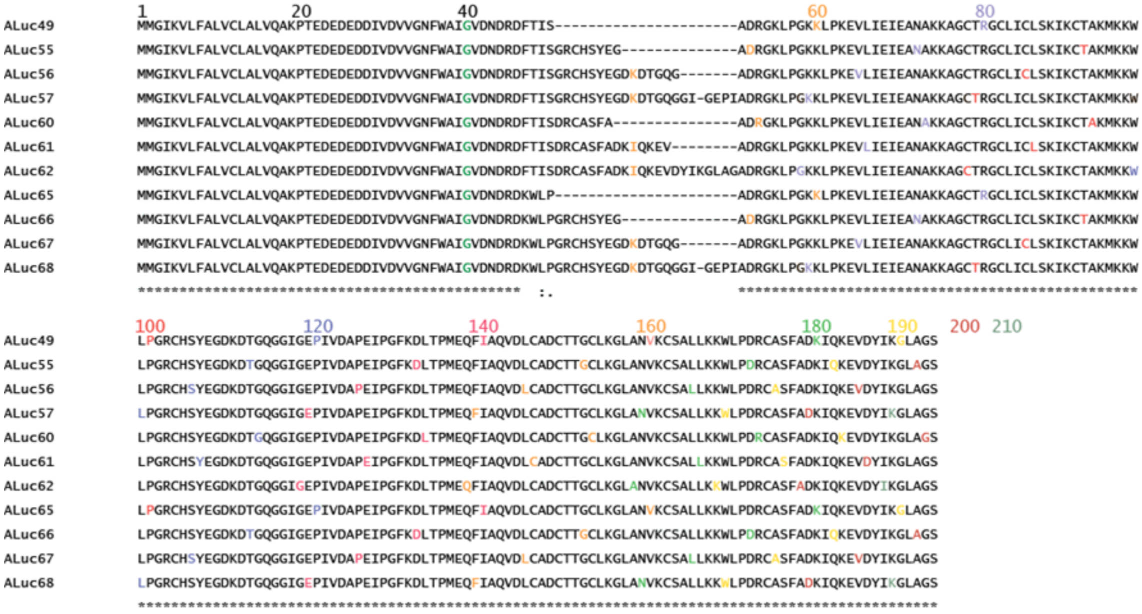

Suppl. Figure S1. Multiple sequence alignment of new Artificial luciferases (ALucs) compared with ALuc49. Every 20th amino acid was highlighted in colors.

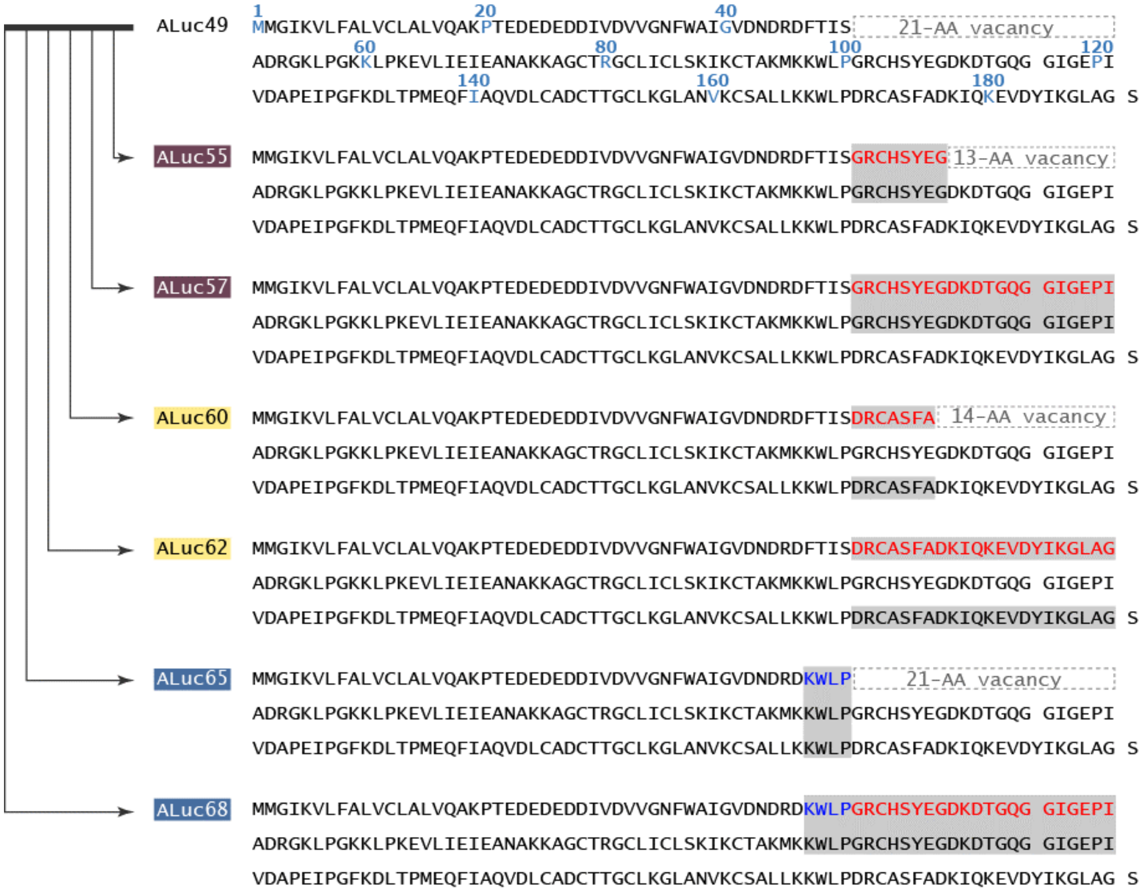

Suppl. Figure S2. Single-sequence alignments (SSAs) of new ALucs to highlight the appended sequences. The gray shadows highlight the characteristic sequence blocks.

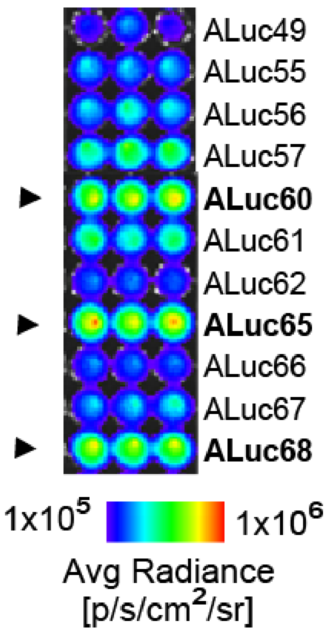

Suppl. Figure S3. The representative BL image of the new ALucs in the presence of native coelenterazine (nCTZ). This is the optical image of Figure 2A.

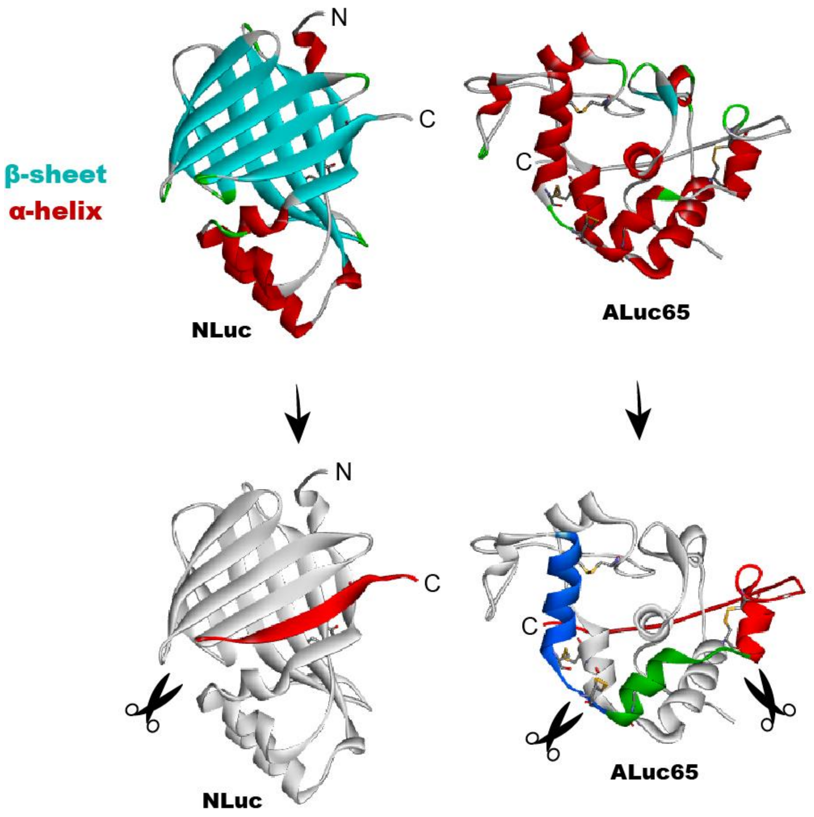

Suppl. Figure S4. Three dimensional structures of NLuc and ALuc65 showing the α-helices and β-sheets. Inspired by the molecular structures, we decided the fragmentation sites for single-chain BL probes. The scissors marks highlight the dissection sites that are located at the hinge regions between α-helices or β-sheets. The structural models of new ALucs (ALuc60–68), which were created using SWISS-MODEL (https://swissmodel.expasy.org) [1]

Suppl. Table S1. Homologous proteins (rankings) of ALuc56, ALuc65, and ALuc68. Those were searched by NCBI BLAST.

ALuc56

Ranking	Name	Accession ID	Identity (%)
1	Synthetic construct (S-14D5α-MLuc7-H7)	QND76010.1	76.4%
2	*Pleuromamma xiphias* luciferase	BAN91830.1	74.8%
3	Synthetic construct (ALuc34)	AYN79601.1	72.8%
4	*Metridia pacifica* luciferase	BAG48249.1	72.5%

ALuc65

Ranking	Name	Accession ID	Identity (%)
1	Synthetic construct (S-14D5α-MLuc7-H7)	QND76010.1	77.2%
2	*Pleuromamma xiphias* luciferase	BAN91830.1	74.8%
3	Hypothetical protein from *Salmonella enterica*	EAU0845767.1	74.4%
4	Hypothetical protein from *Salmonella enterica*	EAQ6767855.1	73.5%

ALuc68

Ranking	Name	Accession ID	Identity (%)
1	*Metridia pacifica* luciferase	BAD93334.1	79.3%
2	Synthetic construct (S-14D5α-MLuc7-H7)	QND76010.1	77.2%
3	*Metridia longa* luciferase 2	APQ47582.1	75.0%
4	*Pleuromamma xiphias* luciferase	BAN91830.1	74.8%

Suppl. Table S2. Relative optical intensity values of the bar graphs in Figure 1D, compared to those of GLuc. The terms, “Luc” and “Sub”, denote marine luciferase and substrate, respectively.

Sub / Luc	nCTZ	1a	1b	1c	1d	2a	2b	2c	2d	3a	3b	3c
**GLuc**	1.0	1.0	1.0	1.0	1.0	1.0	1.0	1.0	1.0	1.0	1.0	1.0
**MLuc**	0.3	1.0	1.2	4.3	1.4	1.1	1.0	1.0	1.0	1.1	1.0	0.9
**RLuc8.6-535**	1.8	81.6	941.9	159.8	22.2	1.4	1.0	1.0	1.0	1.0	1.0	0.8
**ALuc16**	13.6	9.0	5.8	5.4	2.8	1.4	1.1	3.6	1.7	1.1	1.0	1.0
**ALuc23**	5.6	5.3	2.1	2.7	1.4	1.4	1.3	1.2	1.2	1.3	1.2	1.1
**ALuc49**	19.8	11.8	4.8	8.7	2.7	1.5	1.4	1.9	1.4	1.2	1.2	1.0
**ALuc55**	19.7	10.6	4.9	8.8	2.4	1.3	1.3	1.8	1.2	1.1	1.1	0.8
**ALuc56**	26.3	13.1	4.9	8.8	2.5	1.3	1.2	1.9	1.1	0.9	0.9	0.8
**ALuc57**	6.7	2.2	3.8	8.1	1.9	0.9	0.9	2.1	0.9	0.7	0.7	0.7
**ALuc60**	18.4	5.8	8.4	15.5	3.6	1.0	1.0	3.7	1.4	0.8	0.9	0.8
**ALuc61**	11.9	4.4	4.3	7.6	2.0	1.0	0.8	2.0	1.1	0.7	0.7	0.6
**ALuc62**	8.7	4.0	3.0	5.1	1.5	0.8	0.7	1.4	0.7	0.8	0.8	0.6
**ALuc65**	30.9	6.4	9.1	18.7	4.4	1.4	1.5	3.6	1.6	1.1	1.1	0.8
**ALuc66**	9.8	2.8	4.1	8.1	2.3	1.4	1.3	1.7	1.1	1.2	0.7	0.6
**ALuc67**	22.4	5.1	5.8	11.6	2.6	1.3	1.2	2.7	1.2	0.8	0.8	0.6
**ALuc68**	34.5	7.3	6.5	13.4	3.0	1.1	1.2	2.5	1.4	0.8	0.8	0.9

References

1. Waterhouse, A.; Bertoni, M.; Bienert, S.; Studer, G.; Tauriello, G.; Gumienny, R.; Heer, F. T.; de Beer, T. A. P.; Rempfer, C.; Bordoli, L.; Lepore, R.; Schwede, T., SWISS-MODEL: homology modelling of protein structures and complexes. *Nucleic Acids Res.* **2018,** 46, (W1), W296-W303.
